# A Rare but Life‐Threatening Complication of Direct Endoscopic Necrosectomy: Tension Pneumoperitoneum With Pneumothorax and Hypercapnia due to Walled‐Off Necrosis Rupture

**DOI:** 10.1155/crgm/6599334

**Published:** 2026-04-29

**Authors:** Yasuo Otsuka, Kosuke Minaga, Akane Hara, Mamoru Takenaka, Chihoko Nobori, Takaaki Murase, Ippei Matsumoto, Masatoshi Kudo

**Affiliations:** ^1^ Department of Gastroenterology and Hepatology, Kindai University Faculty of Medicine, Sakai, Japan, kindai.ac.jp; ^2^ Department of Surgery, Kindai University Faculty of Medicine, Sakai, Japan, kindai.ac.jp

**Keywords:** acute pancreatitis, endoscopic necrosectomy, hypercapnia, pneumoperitoneum, walled-off necrosis

## Abstract

Endoscopic ultrasonography (EUS)–guided drainage followed by direct endoscopic necrosectomy (DEN) is widely used as a minimally invasive treatment for walled‐off necrosis (WON). While this approach is considered effective and safe, serious complications may occur. We report a case of carbon dioxide (CO_2_)–related tension pneumoperitoneum accompanied by severe hypercapnia during DEN. A 65‐year‐old man with gallstone pancreatitis developed a large WON causing gastric outlet obstruction. He underwent EUS‐guided transgastric drainage with placement of a lumen‐apposing metal stent (LAMS), followed by planned DEN sessions under CO_2_ insufflation with propofol sedation. During the second DEN session, the LAMS became dislodged, and subsequent DEN was continued after balloon dilation of the endosonographically created route. During the fourth DEN session, the patient experienced sudden oxygen desaturation. Imaging revealed extensive free air in the abdominal and thoracic cavities, and arterial blood gas analysis showed severe hypercapnia. Emergency exploratory laparotomy revealed disruption of the WON wall with communication into the abdominal cavity. Surgical necrosectomy and abdominal irrigation were performed. The patient recovered uneventfully. This case highlights a rare but potentially serious CO_2_‐related complication of DEN and underscores the need for meticulous control of intracavitary pressure and close respiratory monitoring during the procedure.

## 1. Introduction

Acute pancreatitis (AP) is most commonly caused by excessive alcohol consumption, gallstones, and metabolic disorders [1]. In a subset of patients with severe AP, ongoing inflammation may lead to the development of walled‐off necrosis (WON) as a late local complication. When WON becomes infected or produces symptoms due to compression of adjacent organs, interventional drainage is indicated. In many cases, a large volume of solid necrotic debris remains within the cavity, making necrosectomy necessary after initial drainage to achieve clinical resolution [2].

In recent years, a step‐up approach has become the standard treatment strategy, in which minimally invasive endoscopic interventions are performed before surgical necrosectomy [3, 4]. Typically, a lumen‐apposing metal stent (LAMS) is placed under endoscopic ultrasonography (EUS) guidance to create a wide and stable fistula between the gastrointestinal lumen and the WON cavity. This allows endoscopic access to the necrotic space and facilities repeated sessions of direct endoscopic necrosectomy (DEN) [5].

DEN has gained widespread acceptance as an effective and minimally invasive treatment for WON. Recent systematic reviews and meta‐analyses have reported favorable clinical outcomes with acceptable safety profiles, with overall adverse event rates that are substantially lower than those associated with surgical necrosectomy [6, 7]. Nevertheless, procedure‐related complications—including intraprocedural or delayed bleeding, perforation, and stent occlusion or migration—remain important clinical concerns. Such complications may be associated with the creation of a large transmural fistula and the extensive manipulation required within a semiclosed necrotic cavity.

Herein, we report a rare but potentially life‐threatening case of carbon dioxide (CO_2_)–related tension pneumoperitoneum accompanied by severe hypercapnia, resulting from rupture of the WON cavity wall during DEN.

## 2. Case Presentation

A 65‐year‐old man was initially treated for gallstone pancreatitis at another hospital. He underwent endoscopic retrograde cholangiopancreatography (ERCP) with endoscopic sphincterotomy followed by biliary stent placement, after which his pancreatitis worsened, raising the possibility that, in addition to gallstone pancreatitis, post‐ERCP pancreatitis may also have contributed to the clinical course. Subsequently, as a local complication of pancreatitis, he developed WON. Progressive enlargement of the WON resulted in symptomatic gastric outlet obstruction, and the patient was referred to our hospital for further management 30 days after the onset of gallstone pancreatitis.

On admission, physical examination revealed a body temperature of 37.0°C, blood pressure of 149/87 mmHg, heart rate of 95 beats/min, and respiratory rate of 18 breaths/min. Laboratory evaluations showed only a minimal elevation in C‐reactive protein (CRP, 8.7 mg/L; reference interval: < 5 mg/L) and a modestly increased serum lipase level (375 U/L; reference interval: 13–60 U/L). The patient complained of persistent nausea accompanied by abdominal fullness and difficulty with oral intake.

**FIGURE 1:**
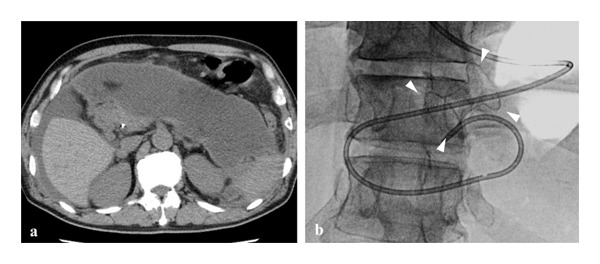
Imaging findings at the time of transfer and initial drainage. (a) Contrast‐enhanced computed tomography at the time of transfer to our institution demonstrating a large walled‐off necrosis extending into the pelvic cavity, compressing the stomach and resulting in gastric outlet obstruction. (b) Fluoroscopic image demonstrating the placement of a lumen‐apposing metal stent (Hot AXIOS, 15‐mm diameter × 10‐mm length) between the stomach and the WON cavity under endoscopic ultrasonography guidance (arrowheads).

Contrast‐enhanced computed tomography (CT) revealed a large WON extending into the pelvic cavity, with marked compression of the stomach (Figure 1(a)). Endoscopic drainage was therefore indicated. On hospital Day 4, EUS‐guided transgastric drainage of the WON was performed, and a LAMS (Hot AXIOS, 15‐mm diameter × 10‐mm length; Boston Scientific, Marlborough, MA, USA) was deployed between the stomach and the WON cavity (Figure 1(b)). Because a substantial amount of necrotic debris remained after initial drainage, planned DEN was initiated. DEN sessions were scheduled twice weekly, and multiple procedures were subsequently conducted. All DEN procedures were carried out under CO_2_ insufflation with propofol sedation using a GIF‐290T endoscope (Olympus Medical Systems, Tokyo, Japan). Supplemental oxygen was routinely administered via nasal cannula at 2 L/min. End‐tidal carbon dioxide (EtCO_2_) was not monitored, and all procedures were performed in the prone position.

At the second DEN session, the LAMS became dislodged during endoscopic manipulation. As a mature endosonographically created route (ESCR) between the stomach and the WON cavity had already formed, balloon dilation of the ESCR was performed using a 14–16 mm dilation balloon (GIGA‐II; Century Medical, Tokyo, Japan). The endoscope was then advanced directly into the WON cavity, and DEN was continued.

**FIGURE 2:**
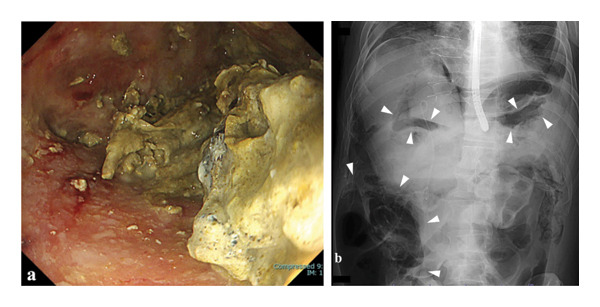
Endoscopic and fluoroscopic findings at the time of cavity rupture during the fourth direct endoscopic necrosectomy. (a) Endoscopic view during direct endoscopic necrosectomy showing the lumen of the WON cavity; no endoscopic findings suggestive of perforation were observed. (b) Fluoroscopic image obtained during the same session demonstrating free intraperitoneal air at multiple sites (arrowheads).

**FIGURE 3:**
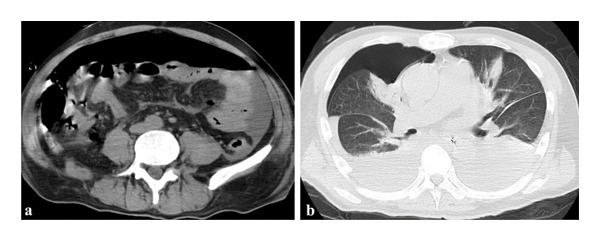
Computed tomography findings after the fourth direct endoscopic necrosectomy. (a) Abdominal computed tomography showing a large amount of free intraperitoneal air. (b) Chest computed tomography demonstrating right pneumothorax.

On hospital Day 20, during the fourth DEN session, balloon dilation of the ESCR was again performed, followed by continuation of DEN (Figure 2(a)). During the procedure, a sudden decrease in oxygen saturation was observed. Fluoroscopic imaging revealed findings highly suggestive of massive intraperitoneal free air (Figure 2(b)). The procedure was immediately discontinued, and the endoscope was withdrawn. Despite this, the patient’s oxygenation did not improve, necessitating emergency endotracheal intubation. During the event, marked abdominal distension consistent with severe pneumoperitoneum was observed, whereas no clear physical examination findings suggestive of tension pneumothorax were noted. Emergent decompression was performed by abdominal needle puncture with an 18‐gauge needle, releasing a large volume of gas. Arterial blood gas analysis obtained after the DEN revealed severe hypercapnia and respiratory acidemia, with a PaCO_2_ of 76.3 mmHg (10.2 kPa; reference interval: 4.7–6.0 kPa) (Table 1). Subsequent CT revealed a large amount of free air in the abdominal cavity and pneumothorax in the chest (Figure 3).

**TABLE 1:** Arterial blood gas analysis obtained after endoscope withdrawal during the fourth direct endoscopic necrosectomy.

Parameter	Value	Reference interval (SI units)
pH	7.118	7.35–7.45
pCO_2_	76.3 mmHg (10.2 kPa)	4.7–6.0 kPa
pO_2_	56.7 mmHg (7.6 kPa)	10.7–13.3 kPa
HCO_3_	24.1 mmol/L	22–26 mmol/L
ABE	−6.3 mmol/L	−2 to +2 mmol/L
SBE	−5.2 mmol/L	−2 to +2 mmol/L

*Note:* pCO_2_, partial pressure of carbon dioxide; pO_2_, partial pressure of oxygen; HCO_3_^−^, bicarbonate.

Abbreviations: ABE, actual base excess; SBE, standard base excess.

In the presence of pneumoperitoneum, pneumothorax, and severe hypercapnia, it was considered that CO_2_ used for insufflation during DEN had leaked into and accumulated within the abdominal cavity as a result of disruption of either the ESCR or the WON cavity, with possible subsequent extension into the thoracic cavity, contributing to impaired ventilation.

**FIGURE 4:**
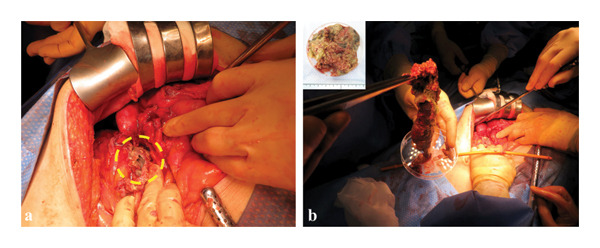
Intraoperative findings during emergency laparotomy. (a) Disruption of the wall of the walled‐off necrosis identified at the right margin of the WON cavity (dashed circle), with direct communication between the WON cavity and the abdominal cavity. (b) Surgical necrosectomy was performed, with removal of a total of 61 g of necrotic tissue from the WON cavity.

Given the patient’s rapidly deteriorating clinical condition, emergency exploratory laparotomy was performed to determine the source of massive intraperitoneal free air. Intraoperatively, turbid ascites was noted within the abdominal cavity; however, no overt perforation of the gastrointestinal tract or the ESCR was identified. Instead, purulent material was observed leaking from the right margin of the WON cavity, and disruption of the WON wall with direct communication into the abdominal cavity was confirmed (Figure 4(a)).

The WON cavity was surgically opened, and necrosectomy was performed, with removal of approximately 61 g of necrotic material (Figure 4(b)), followed by thorough irrigation of the abdominal cavity. Cholecystectomy was performed concurrently to address the underlying etiology of pancreatitis, and drains were placed in both the WON cavity and the abdominal cavity.

Postoperatively, the patient was managed in the intensive care unit (ICU). His postoperative course was complicated by bile leakage after cholecystectomy, requiring prolonged respiratory support with tracheostomy. He was transferred out of the ICU on postoperative Day 34 while still requiring mechanical ventilation and was successfully weaned from ventilatory support on postoperative Day 47. All surgical drains were removed on Day 81, and he was discharged home on postoperative Day 98. During more than 1 year of follow‐up, the patient has remained free of recurrent pancreatitis or WON and has fully returned to normal daily activities.

## 3. Discussion

A previous meta‐analysis demonstrated that metal stents achieve significantly higher clinical success rates than plastic stents for endoscopic drainage of WON [8]. With the introduction of LAMSs, EUS‐guided drainage followed by DEN has become a widely adopted first‐line, minimally invasive approach for the management of WON [3]. Nevertheless, despite its minimally invasive nature, DEN is associated with a nonnegligible risk of procedure‐related complications. According to the Asian consensus statements, perforation has been reported in 28 of 633 cases (4.4%) [9]. In this context, we encountered a rare but severe complication in which rupture of the WON wall during DEN resulted in tension pneumoperitoneum and profound hypercapnia.

One plausible mechanism underlying this complication relates to the procedural environment of DEN. In this case, DEN was performed under propofol sedation with CO_2_ insufflation within a semiclosed necrotic cavity. Repeated cycles of insufflation and suction within a confined space, together with repeated endoscopic manipulation during DEN, guidewire exploration of the cavity, and balloon dilation of the ESCR, may increase intracavitary pressure and mechanical stress on the fragile wall of the WON. Under such conditions, disruption of the cavity may occur, allowing insufflated CO_2_ to escape into the abdominal cavity. The gas may subsequently extend into the thoracic cavity, possibly through microscopic diaphragmatic defects, resulting in pneumothorax. In addition, because CO_2_ is rapidly absorbed into the systemic circulation and pneumothorax may further impair ventilation, these processes can cause abrupt and severe hypercapnia. In this context, pneumothorax likely played a major role in the development of severe hypercapnia, rather than pneumoperitoneum alone.

In addition to this general mechanism, technical factors related to device selection may have influenced intracavitary pressure dynamics in this case. Initially, a 15‐mm LAMS was placed, and DEN was performed using a GIF‐H290T endoscope (outer diameter, 9.8 mm). Under these conditions, the diameter difference between the LAMS and the endoscope likely provided sufficient space for CO_2_ to escape from the WON cavity into the stomach, thereby limiting excessive intracavitary pressure during DEN (Figure 5(a)). After dislodgement of the LAMS, however, DEN was continued following balloon dilation of the ESCR to 14–16 mm. In the absence of a LAMS, balloon dilation alone may not maintain a stable and consistently wide access diameter. Consequently, the effective difference between the access diameter and the endoscope diameter may have been reduced, impairing CO_2_ escape during DEN. Under these circumstances, continued CO_2_ insufflation may have led to a progressive increase in intracavitary pressure, ultimately contributing to rupture of the WON wall (Figure 5(b)).

FIGURE 5Schematic illustration of the hypothesized mechanism of WON cavity rupture. (a) When a lumen‐apposing metal stent (LAMS) is in place, carbon dioxide insufflated during endoscopic necrosectomy can escape into the stomach through the space between the endoscope and the LAMS, thereby limiting intracavitary pressure elevation. (b) After LAMS dislodgement, the interface between the endoscope and the surrounding mucosa may function as a check‐valve mechanism, impairing gas escape. As a result, intracavitary carbon dioxide pressure may increase, potentially leading to rupture of the fragile WON wall.

**Figure:**
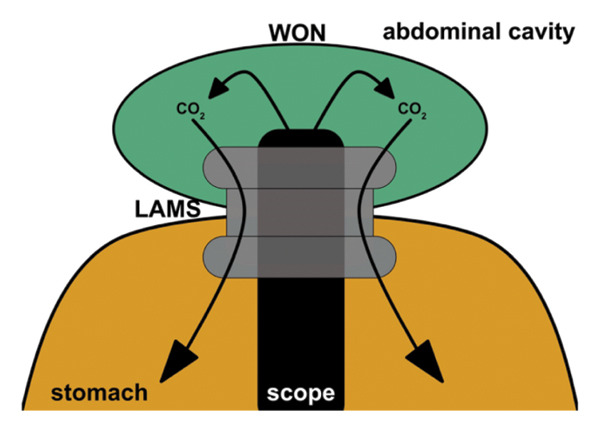
(a)

**Figure:**
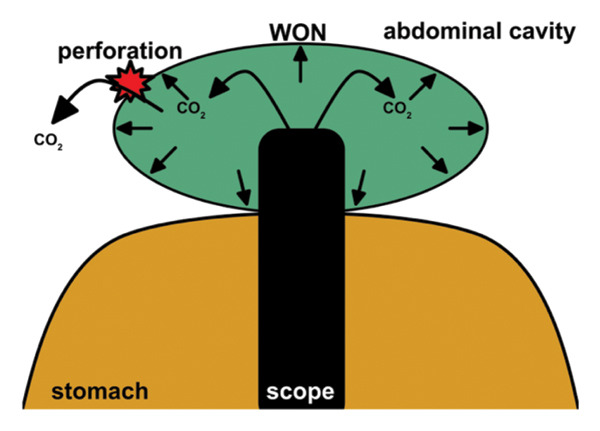
(b)

Therefore, the creation of a sufficiently wide and stable access route is important for intracavitary pressure control during DEN. The use of a larger‐diameter LAMS or performance of DEN with a smaller‐caliber endoscope may increase the available working space, facilitate more effective CO_2_ escape, and potentially reduce intracavitary pressure and the risk of WON wall disruption.

Although this consideration remains speculative, device selection and the balance between access diameter and endoscope size may represent important technical factors for the safe performance of DEN. Moreover, while LAMS dislodgement is not an uncommon adverse event during DEN [10], redeployment of a LAMS before continuing the procedure may enhance procedural safety by reestablishing a stable and adequately wide access route.

Early recognition of the complication is also critical. In our case, the event was recognized following a sudden decrease in oxygen saturation; however, hypoxemia is generally considered a relatively late manifestation of ventilatory compromise. Previous studies have shown that EtCO_2_ monitoring can detect ventilatory abnormalities and CO_2_ retention earlier than pulse oximetry during endoscopic procedures performed under sedation, including those using CO_2_ insufflation [11, 12].

Furthermore, current sedation guidelines recommend capnography, particularly during prolonged or high‐risk endoscopic interventions, to facilitate early detection of respiratory depression [13]. Given that DEN is frequently performed in a closed or semiclosed space under CO_2_ insufflation, EtCO_2_ monitoring may allow earlier recognition of CO_2_ retention and impending respiratory compromise before the onset of oxygen desaturation. Although continuous EtCO_2_ monitoring was not available during DEN in this case, its potential value for the early detection of ventilatory compromise during DEN should be noted.

In addition to physiological monitoring, imaging may also contribute to early recognition of this complication. Although fluoroscopy is not routinely emphasized during DEN, cases of pneumoperitoneum caused by perforation of the necrotic cavity wall during DEN have been reported [14]. In such situations, intermittent assessment of fluoroscopic images for unexpected or abnormal gas distribution may facilitate earlier recognition of cavity rupture or gas leakage, particularly when clinical or ventilatory changes are observed.

In conclusion, this case illustrates a rare but severe insufflation‐related complication during DEN following LAMS dislodgement. Even after apparent maturation of the ESCR, alterations in CO_2_ egress dynamics may lead to dangerous elevations in intracavitary pressure. Early recognition of hypercapnia and related clinical signs, together with careful insufflation management and appropriate device selection, is essential when continuing DEN after LAMS dislodgement.

## Author Contributions

Y.O. and K.M. conceived the study and drafted the manuscript. Y.O., K.M., A.H., C.N., and T.M. were involved in the clinical management of the patient. A.H., C.N., T.M., and M.T. contributed to critical revision and editing of the manuscript. I.M. and M.K. supervised the study.

## Funding

This work was supported by a Grant‐in‐Aid for Scientific Research (25K19310) from the Japan Society for the Promotion of Science and a Kindai University Research Enhancement Grant (IP007).

## Disclosure

All authors have read and approved the final manuscript.

## Ethics Statement

This study was conducted in accordance with the ethical principles of the Declaration of Helsinki for medical research involving human participants.

## Consent

Written informed consent was obtained from the patient for the publication of this case report and any accompanying images.

## Conflicts of Interest

The authors declare no conflicts of interest.

## Data Availability

The data supporting the findings of this case report are available from the corresponding author upon reasonable request.
